# In Silico Study of Potential Binding Sites of the Family GH126 Enzyme CPF_2247 from *Clostridium perfringens* Using Structural Comparison and Molecular Docking Methods

**DOI:** 10.3390/molecules31132273

**Published:** 2026-06-29

**Authors:** Michaela Hodorová, Štefan Janeček

**Affiliations:** 1 Laboratory of Protein Evolution, Institute of Molecular Biology, Slovak Academy of Sciences, SK-84551 Bratislava, Slovakia; michaela.hodorova@savba.sk; 2 Department of Biology, Institute of Biology and Biotechnology, Faculty of Natural Sciences, University of SS. Cyril and Methodius, SK-91701 Trnava, Slovakia

**Keywords:** family GH126, CPF_2247, structural comparison, molecular docking

## Abstract

The family GH126 represents a potential fourth, but still non-confirmed α-amylase family in CAZy with the founding, partially characterized member, the assumed amylolytic enzyme CPF_2247 from *Clostridium perfringens*. Proteins of this family adopt an (α/α)_6_-barrel domain, structurally distinct from the rather more complex domain arrangement of families GH13, GH57, and GH119. Interestingly, GH126 exhibits structural similarity, including sharing potential functionally important residues with inverting β-glucanases from GH8 and GH48 (clan GH-M); this fact has prompted previous bioinformatics analyses. In the present study, two GH126 members with experimentally determined tertiary structure—the CPF_2247 and the exopolysaccharide-specific hydrolase PssZ from *Listeria monocytogenes*—were compared with seven GH8 and ten GH48 enzyme-substrate complexes. Family GH126 enzymes display a wide, open binding cleft, with a central tunnel-like cavity along the barrel axis, distinct from the narrow cleft in GH8 and the tunnel-shaped site in GH48. Conserved residues involved in substrate binding and catalysis of GH8 and GH48 were identified in GH126. Molecular docking with α-glucans using the CPF_2247 confirmed predicted binding at the potential active site and revealed also eventual additional binding sites. Targeted docking showed the strongest interactions for acarbose and maltoheptaose, particularly involving a GH126 unique α11-α12 loop in the assumed amylolytic enzyme CPF_2247.

## 1. Introduction

The glycoside hydrolase (GH) family 126 was established in the Carbohydrate-Active Enzyme Database (CAZy; [1]; https://www.cazy.org/, accessed on 21 November 2024) in 2011 based on the determination of the three-dimensional structure of the CPF_2247 protein of *Clostridium perfringens* [2]. Although it exhibited a pronounced structural homology to β-glucanases from the CAZy clan GH-M, i.e., the families GH8 and GH48 [3,4], its low sequence similarities to members of both families of the clan GH-M and the observed amylolytic activity led to the creation of a new family, the family GH126 [2]. Unfortunately, the obvious discrepancy between the structural homology to β-glucan-active glycosidases and declared activity of an α-glucan-active enzyme (an amylase), together with some inconsistency concerning the endo- vs. exo-amylolytic mode of action [2], has made the knowledge of exact enzymatic specificity present in the family GH126 rather unclear [5,6]. In fact, in addition to the fundamental study by Ficko-Blean et al. [2], there is only one more partially characterized GH126 member, the protein PssZ from *Listeria monocytogenes* [7]. Its three-dimensional structure has also been determined [8]. However, in contrast to CPF_2247, the PssZ has been characterized as an exopolysaccharide (EPS) specific GH; the EPS being formed by the N-acetylmannosamine and galactose in a ratio 2:1 [7].

Although the founding member of the family GH126, the protein CPF_2247 of *C. perfringens*, has been proposed to be an amylolytic enzyme (even potentially an α-amylase [1,2]), the supposed catalytic (α/α)_6_-barrel catalytic domain differs from the catalytic fold present in both α-amylase families GH13 and GH57 [7,8]. While in the former, there is the classical (β/α)_8_-barrel (the TIM barrel) [7,9,10], the members of the latter adopt the incomplete seven-stranded version of the TIM barrel [7,11,12]. The same applies to their clans GH-H and GH-T, respectively. Moreover, all of them employ the retaining mechanism for cleaving the α-glucosidic linkages [1]. Also, β-amylases classified in the family GH14 possess the catalytic TIM-barrel [13,14]. They use the inverting reaction mechanism [1]. Only glucoamylases of the family GH15 employing the inverting mechanism [15,16] share the (α/α)_6_-barrel fold [15,16] with GH126 members. But a much more pronounced similarity of the GH126 (α/α)_6_-barrel has been revealed to that present in retaining α-mannanases from the family GH76 [17]. This might be of special interest, since GH76 members are α-glucan-active enzymes [1]. The efforts aimed to shed some additional light on substrate preference and product profile of the CPF_2247 protein and its GH126 counterparts are thus extremely required. Without further experiments, however, their sequence-structural homology to β-glucan-active inverting enzymes from families GH8 and GH48 (i.e., the clan GH-M), identified originally [2], has still to be considered [18]. In fact, nothing conclusive is known in the family GH126 concerning either the catalytic machinery, active-site architecture, exact enzyme specificity, or even the employed reaction mechanism [2,5,6,7,8,17,18,19].

Nevertheless, three detailed bioinformatics analyses have been performed so far [17,18,19]. The first one [17] delivered mainly the establishment of seven conserved sequence regions (CSRs) in amino acid sequences of family GH126 proteins and their basic evolutionary relationships, including also identifying the above-mentioned remotely related (α/α)_6_-barrel homologs in the family GH76 [20]. Since the family GH126 has been, from its beginning in 2011, recognized as the prokaryotic family with proteins originating solely from the phylum Bacillota [1], the second in silico work [19] successfully expanded the scope of the family outside Bacillota, but still did not find any potential protein outside the prokaryotes. Finally, the third bioinformatics study [18] identified unique sequence-structural features in GH126 CSRs (the sequence logo based on 1665 sequences), warranting the correct assignment of new hypothetical proteins just to family GH126, but not to either GH8 or GH48. Moreover, two eukaryotic GH126 members were clearly traced among the fungi, and a group of the so-called intermediary proteins, i.e., a group between the family GH126 and the clan GH-M (GH8 and GH48), was observed [18].

The main goal of the present study was therefore to contribute to the still unsolved question concerning the enzyme activity and specificity of GH126 members. This was achieved first by a detailed comparison of both GH126 members with the three-dimensional structure already determined, the CPF_2247 from *C. perfringens* and the PssZ from *L. monocytogenes*, with relevant representatives of both families of the clan GH-M, focusing especially on those GH8 and GH48 members for which their tertiary structure has been solved as a complex. Then, various α-glucans, as well as recognized substrates of amylolytic starch hydrolases and related enzymes, were used in docking trials with the GH126 structure of the assumed amylolytic enzyme CPF_2247 executed as both blind and targeted experiments.

## 2. Materials and Methods

### 2.1. Searching for GH126 Structural Homologs

To identify structurally related proteins to family GH126 members, the Foldseek server ([21]; https://search.foldseek.com/search, accessed on 16 October 2024) was used to search against experimentally determined structures in the Protein Data Bank (PDB; [22]) using the default 3Di/AA mode, which combines three-dimensional structural information with traditional amino acid sequence data. The search was performed with the two GH126 experimentally determined structures: (i) the CPF_2247 from *C. perfringens* ([2]; PDB code: 3REN); and (ii) the PssZ from *L. monocytogenes* ([7]; PDB code: 6R2M). Both were retrieved from (PDB; [22]; https://www.rcsb.org/, accessed on 16 October 2024) and used as full-length structures, each comprising a single catalytic (α/α)_6_-barrel domain. The identified proteins were subsequently classified into GH families according to CAZy ([1]; https://www.cazy.org/, accessed on 1 November 2024). In cases where CAZy annotation was not available, classification was completed using the entries in the InterPro database ([23]; https://www.ebi.ac.uk/interpro/, accessed on 14 November 2024), which recently includes also the Pfam database [24].

### 2.2. Structural Comparison of GH126 with GH8 and GH48 Enzyme-Ligand Complexes

In addition to representatives of the family GH126, the CPF_2247 from *C. perfringens* [2] and the PssZ from *L. monocytogenes* [7], three-dimensional structures determined as complexes with respective ligands of seven GH8 and ten GH48 enzymes (Table 1) were retrieved from PDB ([22]; https://www.rcsb.org/, accessed on 20 November 2024).

When multiple structures were available for a given enzyme, preference was given to those containing longer oligosaccharide ligands spanning multiple subsites and to structures determined at higher resolution. Information on enzyme specificity was obtained from CAZy ([1]; https://www.cazy.org/, accessed on 21 November 2024) and relevant literature.

Structural superpositions were performed using the UCSF ChimeraX ([41]; version 1.10) and its “matchmaker” tool applied with the “best-alignment pair of chains” option. For visualization, the program PyMOL ([42]; version 2.5.7) was used.

The sequence-based alignment taken as a reference was derived from a previous in silico analysis of 434 sequences from all three families, GH126, GH8, and GH48 [18].

Interactions between ligands and active-site residues were analyzed using UCSF ChimeraX [41], PyMOL [42], and LigPlot+ [43] with support of data extracted from published literature [2,3,4,5,6,18,25,26,27,28,29,30,31,32,33,34,35,36,37,38,39]. First, LigPlot+ was used to generate two-dimensional interaction diagrams, while hydrogen bonds, distances, and geometric parameters were manually inspected from UCSF ChimeraX and PyMOL. Then, the achieved results were cross-checked by published data on functionally important residues in both GH8 and GH48 families, as well as with respect to knowledge of GH126.

### 2.3. Molecular Docking

Molecular docking trials were performed using the AutoDock Vina ([44]; version 1.1.2), as implemented in UCSF Chimera ([45]; version 1.18). The receptor structure, i.e., the assumed amylolytic enzyme CPF_2247 from *C. perfringens* ([2]; PDB code: 3REN), was obtained from PDB [22]. For docking, the structure was modified to include only chain A, based on monomeric assembly predictions obtained using PDBePISA ([46]; https://www.ebi.ac.uk/pdbe/pisa/, accessed on 3 December 2024). Prior to docking, in order to avoid interference with ligand binding site identification, all non-protein molecules were removed from the structure, including crystallographic water molecules, sulfate ions, ethylene glycol, and magnesium ions.

Docking calculations were carried out using a rigid receptor and flexible ligands. Using a rigid receptor should be justified since, e.g., in the related family GH8, no substantial conformational changes were observed in tertiary structure, regardless of whether it was solved in the apo-form without any ligand or if determined as a complex with a ligand [26]. Output files were generated in PDBQT format and analyzed in UCSF ChimeraX [41] using the ViewDockX tool. Binding energy scores, hydrogen bonding interactions, and steric clashes were evaluated for each predicted pose. To ensure reproducibility, docking calculations were performed using an exhaustiveness value of 8, with an energy range of 3 kcal/mol and a maximum of 9–10 binding modes generated per ligand.

As ligands, ten α-glucans were selected (Table 2). They were retrieved from the PubChem database ([47]; https://pubchem.ncbi.nlm.nih.gov/) and converted into PDB coordinates by the SMILES program ([48]; https://cactus.nci.nih.gov/translate/, accessed on 12 December 2024). No additional geometry optimization of the ligand structures was performed prior to docking.

Preparation of the receptor (the CPF_2247 protein) and ligands included removal of water molecules, addition of hydrogen atoms, and assignment of Kollman partial charges using the UCSF Chimera [45]. Ligand structures were treated as flexible during docking. The docking protocol was previously validated using a reference protein-ligand complex with a known experimental structure, indicating that the employed ligand preparation procedure was sufficient to reproduce biologically relevant binding modes. Where applicable, protonation states were assigned to reflect physiological conditions. Docking results were evaluated based on binding energy score, interaction geometry, and spatial localization of ligands relative to the predicted active site.

#### 2.3.1. Blind Docking

Blind docking was performed individually for each ligand. The grid map, set around a search box with dimensions of 51.55, 48.25, 55.60 Å and center coordinates (44.14 × 42.99 × 23.97) Å, was defined to cover the entire enzyme surface. Docking was carried out using the following parameters: exhaustiveness = 8, energy range = 3 kcal/mol, and maximum number of binding modes = 9.

Based on the lowest-energy binding poses identified for each ligand, potential binding sites were determined. The results were subsequently used to define a refined search space corresponding to the putative active-site cleft.

#### 2.3.2. Targeted Docking

Targeted docking was also performed for each ligand using the unified search box centered at (49.99, 45.02, 6.48) Å with dimensions of 45.17 × 31.70 × 42.28 Å. The box position was visually validated in UCSF Chimera [45] to ensure complete coverage of the binding cavity. This grid map encompassed the entire assumed active-site cleft, with an additional margin of approximately 4 Å to allow for ligand flexibility during docking.

Calculations were performed using an exhaustiveness value of 8, an energy range of 3 kcal/mol, and a maximum of 10 binding modes per ligand.

## 3. Results and Discussion

The assumed amylolytic enzyme CPF_2247 from *Clostridium perfringens*, the founding member of the GH126 family [1], adopts an (α/α)_6_ barrel structure of its potential catalytic domain sharing the correspondences with key catalytic and functional residues of the β-glucan-active inverting members of families GH8 and GH48 [2]. According to the original study, on which the family GH126 has been established, the CPF_2247 should cleave amylose and glycogen predominantly by the endo-type of hydrolysis, while for maltooligosaccharides (maltopentaose and larger) it should work using the exo-mode, cleaving the chain by single glucose units. Such a combined activity was interpreted as a consequence of the occupancy of the plus-binding subsites by smaller substrates, which may lead to unproductive complexes or asymmetric cleavage [2]. The alternation of endo- and exo-modes depending on the type of α-glucan substrate is rather a non-standard manifestation of amylolytic activity that—together with observed sequence-structural homology with inverting β-glucanases—causes confusion in efforts to correctly assign the enzyme specificity to GH126 members [8,9] and thus needs to be further understood at the structural level. The performed analyses therefore included: (i) searching for structural homologs of the two characterized GH126 members with already determined tertiary structures; (ii) a detailed structural comparison of both GH126 members with representatives of the two closely related families GH8 and GH48; and (iii) molecular docking trials—both blind and targeted ones—of the CPF_2247 protein with selected α-glucans.

### 3.1. Identifying Structural Homologs

The main aim of the efforts to identify the GH126 structural homologs was to verify previous findings concerning the family GH126 relatedness to inverting β-glucanases of the clan GH-M, i.e., families GH8 and GH48 [2,18]. Furthermore, to reveal any novel, previously undocumented structural relationships. For the analysis, two experimentally determined three-dimensional structures were used: (i) the assumed amylolytic enzyme CPF_2247 from *C. perfringens* ([2]; PDB code: 3REN); and (ii) the EPS-specific hydrolase PssZ from *L. monocytogenes* ([6]; PDB code: 6R2M). Searching through the PDB using the FoldSeek [21] identified a total of 460 and 441 structural hits for the CPF_2247 and PssZ, respectively. In order to capture also more distant structural homologs, no similarity threshold was applied. The highest E-values (i.e., the least significant hits) were found to be 1.56 × 10^−7^ (score 221) for CPF_2247 and 2.36 × 10^−5^ (score 168) for PssZ. For each of the two queries, the top 50 hits with the lowest E-values were selected and ranked according to their FoldSeek score (Table S1).

Non-surprisingly, the lowest E-values were recorded for members of the family GH8; this fact has been well-known from previous observations of structural similarity of GH126 and GH8 [2,6]. For the CPF_2247, the best GH8 scoring hits ranged from 1.5 × 10^−12^ to 1.92 × 10^−8^, while for the PssZ, the values varied from 4.81 × 10^−9^ to 5.36 × 10^−8^. These low E-values clearly point to a highly conserved (α/α)_6_-barrel domain architecture shared by both families GH126 [2,6] and GH8 [3,25,26,27,28,29,30].

On the other hand, despite the definitively confirmed relatedness of the family GH48 not only to GH8 (both are in the clan GH-M [1]) but also to GH126 [2,7,8,18], the FoldSeek search did not assign the GH48 members to hits with a higher scoring (Table S1). This is likely because the overall arrangement of the catalytic (α/α)_6_-barrel in GH48 is more complex than that in both GH126 and GH8, including extended loops and additional β-strands [4,31,32,33,34,35,36,37,38,39]. Since the FoldSeek algorithm assesses, in fact, a global similarity between corresponding protein structures [21], enzymes from the family GH48 appeared among the weaker hits, despite the sequence similarities observed mainly in the seven CSRs throughout all GH126, GH8, and GH48 [17,18,19].

The above approach implemented in the FoldSeek [21] was most probably responsible for repeatedly finding the members of the so-called AGE superfamily (N-acylglucosamine-2-epimerases; Pfam PF07221; [24]) among the high-scoring structures (Table S1). The AGE superfamily covers carbohydrate epimerases and isomerases adopting the catalytic (α/α)_6_-barrel domain with two catalytic histidine residues [49]. As the FoldSeek prioritizes global structural similarities, and the (α/α)_6_-barrel forming the structure of families GH126 and GH8, as well as the AGE superfamily, is compact and not enlarged by other additional structural elements, as it is in family 48, the members of the AGE superfamily [50,51,52,53,54,55] ranked among the hits with relatively high scores. However, further sequence and structural analyses did not reveal in AGE members any significant agreement with the GH126 functionally relevant regions, e.g., seven well-established CSRs [17].

As additional distant structural homologs of GH126, CAZy families GH178, GH76, GH77, GH105, GH88, GH63, GH37, and GH38 (in that order) were identified by FoldSeek (Table S1). Of them, previously only the family GH76 with α-glucan-active members employing the retaining mechanism was found remotely similar to GH126 [17]. However, in the present study, the E-values of GH76 putative endo-α-1,6-mannanase (BT3782) from *Bacteroides thetaiotaomicron* ([56]; PDB code: 4MU9) for both CPF_2247 (4.11 × 10^−7^) and PssZ (4.81 × 10^−5^) represent rather less convincing hits. It is of note that E-values and scores for all GH families (except for GH8 and GH48) were significantly higher and lower, respectively. Although these results indicate some structural similarities in domain arrangement, the probability of true relatedness of those GH families is relatively low.

### 3.2. Structural Comparison of GH126 with GH8 and GH48 Enzyme-Ligand Complexes

Due to the lack of solid experimental data on structure/function relationships in the family GH126 [1,7], the two GH126 representatives with solved tertiary structure—the assumed amylolytic enzyme CPF_2247 [2] and the EPS-specific hydrolase PssZ [5,6]—were structurally compared with members of the most closely related GH families in CAZy, the families GH8 and GH48 forming the clan GH-M [1,2,3,4]. The tertiary structure comparison was performed in an effort to shed some more light on what has been known as yet from the only available and only partially experimentally characterized GH26 member—the CPF_2247 [2]. The gained experimental knowledge that concerns the anticipated mechanism and predicted functionally important residues has been supported so far by a few in silico studies [17,18,19]. The structural comparison should then be crucial for as correct an interpretation as possible of subsequent molecular docking trials.

For comparison purposes, the protein structures of GH8 and GH48 members solved as complexes were selected (Table 1). Both families share the (α/α)_6_-barrel fold and catalytic glutamic acid acting as the proton donor—Glu95 in the GH8 endo-β-1,4-glucanase CelA from *Acetivibrio thermocellus* [3] and Glu87 in the GH48 cellobiohydrolase CelS from *Acetivibrio thermocellus* [4]. Despite the common inverting reaction mechanisms employed in both GH8 and GH48 families, particular details in the mechanism of action differ from each other. Members of GH8, especially cellulases, act mostly by the endo-mode [3,25,26,27,57]; although some exo-xylanases have also been described [28,29]. Cellulases of the family GH48 use the exo-mode primarily [4,31,34,39,57], some being processive [33,36] and even endo-glucanases [32]. These differences are also reflected in the architecture of the active site: GH8 enzymes have a long, open acidic cleft, while the GH48 active-site consists of a predominantly aromatic tunnel opening into a shorter cleft [3,4,25,26,27,28,29,30,31,32,33,34,35,36,37,38,39].

Although the family GH126 contains some of the residues that are functionally important for the clan GH-M [2,18], the shape of the putative active site in GH126 differs significantly from both GH8 and GH48—it is formed by a wide (~17 Å) open cleft that may indicate a different substrate preference.

The set of GH8 and GH48 representatives (Table 1) was therefore collected to cover sufficient information on functionally important residues responsible not only for catalytic action, but also involved in interaction with substrates, forming the overall architecture of the individual active sites. Besides the different enzyme specificities present in both families, one GH48 structure ([35]; PDB code: 5VMA) without data concerning the exact specificity was also taken into account.

The results from the individual structural superpositions of both GH126 representatives with selected members of the clan GH-M are shown in Table 3. While the number of corresponding Cα-atoms varied from 196 to 275 for the CPF_2247 ([2]; PDB code: 3REN; 337 residues) and from 213 to 269 for the PssZ ([6]; PDB code: 6R2M; 317 residues), the root mean square deviation (RMSD) values ranged between 1.73 and 2.21 Å for the CPF_2247 and between 1.88 and 2.15 Å for the PssZ. Overall, however, the superposition results of both GH126 members with GH48 representatives seem to be better, at least when the higher numbers of overlapped corresponding Cα-atoms are concerned. Since all the results were basically comparable to each other (Table 3), it did not make much sense to try to speculate on ascribing any hypothetical specificity of either GH126 enzymes mimicking the determined specificity of a GH8 or GH48 member from a particular comparison.

**Figure 1:**
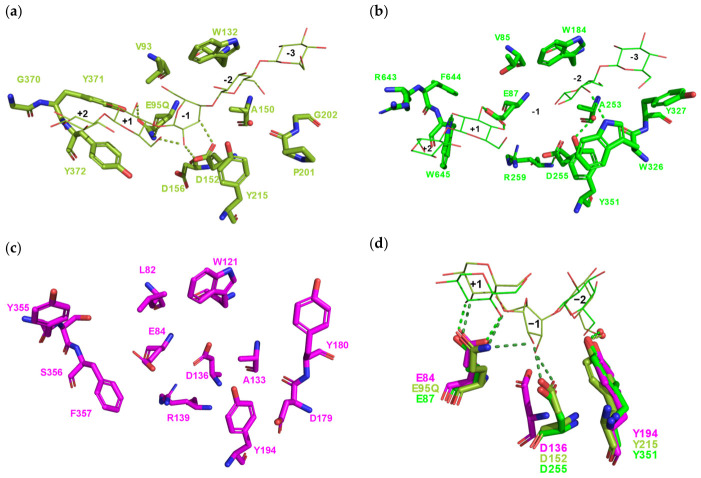
Structural comparison of residues located from the −3 to +2 subsites in families GH8 and GH48 with the corresponding residues of the family GH126. Residues of interest highlighted in the structure of: (**a**) the GH8 endo-β-1,4-glucanase CelA ([3]; PDB code: 1KWF); and (**b**) the GH48 cellobiohydrolase CelS ([4]; PDB code: 1L2A). (**c**) Corresponding residues highlighted in the structure of the GH126 assumed amylolytic enzyme CPF_2247 ([2]; PDB code: 3REN). (**d**) Structural superposition of corresponding functionally important residues: glutamic acids (the general acid catalyst—proton donor in GH8 and GH48), aspartic acids (the substrate recognition and binding site in GH8; the general base—catalytic nucleophile in GH48), and tyrosines (involved in nucleophilic water coordination in GH8 and GH48). Residues of GH126, GH8, and GH48 are coloured magenta, Martian green, and green, respectively. Catalytic water molecules are shown as red spheres, and hydrogen bonds are indicated by dashed lines. Bound cellooligosaccharide ligands from the complexes of GH8 and GH48 structures are also displayed.

A detailed view of the active-site residues complexed with respective cellooligosaccharide ligands occupying the subsites from –3 to +2 for the representatives of GH8 (Figure 1a) and GH48 (Figure 1b) clearly demonstrates their structural similarity to family GH126 represented by the assumed amylolytic enzyme CPF_2247. In fact, the CPF_2247 (Figure 1c) possesses correspondences with most of the residues playing important functional and structural roles in both GH8 and GH48, including the catalytic machinery. Nevertheless, the aspartate (the Asp136 in GH126 CPF_2247) corresponding with the substrate recognition and binding site of GH8 and catalytic nucleophile in GH48 (the Asp 152 and Asp255 in GH8 CelA and GH48 CelS, respectively [58,59]) is in the family GH126 shifted in the amino acid sequence by one position to the N-terminus (Figure 1d). Although the Glu84 can act as a general acid catalyst in GH126, unfortunately, none of the docked ligands were in a favorable position for actual catalysis. Then, it is a hydrophobic leucine (Leu322) that occupies in the CPF_2247 the position corresponding to the general base in GH8 (Asp255 in CelS). Finally, that leucine is located at the helix α11 with its side-chain directed out of the potential active-site cleft. This structural difference could be one of the critical differences between the family GH126 and families GH8 and GH48 of the clan GH-M.

It is, however, worth mentioning that most but not all of the putative GH126 active-site residues identified by this detailed structural comparison with the clan GH-M are present in the seven CSRs established in the GH126 by recent in silico approaches [17,18,19]. Regardless of the Asp136 role in the family GH126, such a phenomenon does not necessarily need to be so rare. For example, in the main α-amylase family GH13, there is the amylolytic enzyme BmaN1 from *Bacillus megaterium* (from the subfamily GH13_45), which may contain an aberrant catalytic machinery [60]. It’s assumed catalytic nucleophile (aspartic acid) is shifted one position to the C-terminus, the position of the family GH13 catalytic site being occupied by a conserved lysine.

### 3.3. Blind-Docking Trials

Following the results achieved by a detailed comparison of three-dimensional structures of the assumed amylolytic enzyme CPF_2247 and EPS-specific hydrolase PssZ from GH126 with those representing the families GH8 and GH48 (clan GH-M), molecular docking trials were undertaken. Molecular docking represents a useful approach, especially if real, i.e., experimentally determined protein and/or enzyme structures solved as complexes with their ligands are still unavailable [61,62].

**Figure 2:**
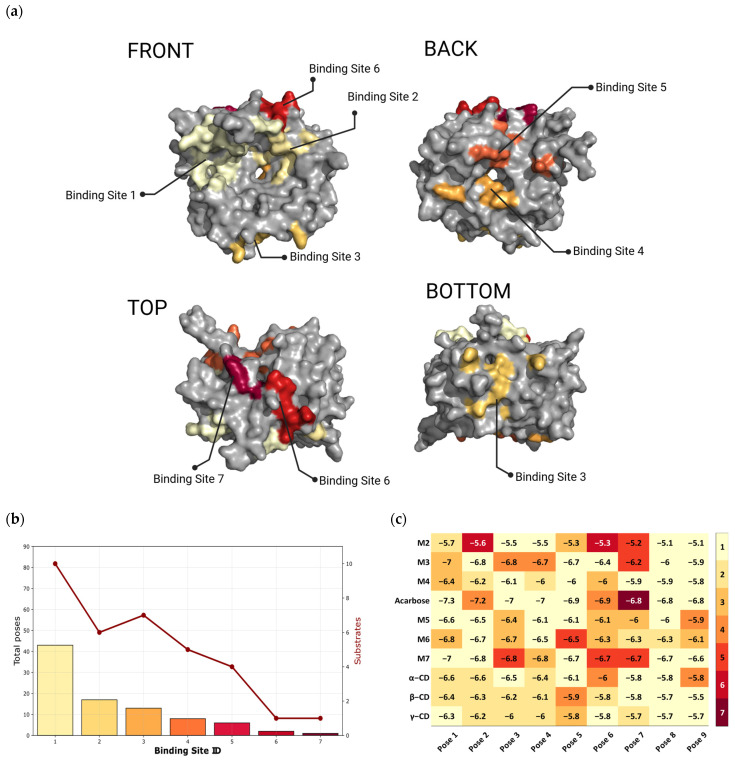
Blind docking identified seven potential binding sites in the assumed amylolytic enzyme CPF_2247. (**a**) Surface representation of CPF_2247 ([2]; PDB code: 3REN) shown from four orientations: front, back, top, and bottom views. The seven potential binding sites are indicated and color-coded. The first two binding sites—BS-1 and BS-2—are proposed as those corresponding to the predicted active-site region. (**b**) Graphical representation of the distribution of docking poses and the number of ligands clustered within each predicted binding site (red line). (**c**) Binding energy scores (kcal/mol) of ligands associated with the individual binding sites.

First, the blind docking was performed to identify all potential binding sites for oligosaccharides (α-glucans) on the protein surface. For docking, only the CPF_2247 [2] was used. Overall, nine set binding modes and ten selected ligands (Table 2) resulted in a total of 90 docking poses. Of them, after clustering, seven distinct binding regions representing potential binding sites (BS; Figure 2) were observed; the most and the least frequently occupied being the BS-1 and BS-7 (eventually also the BS-6), respectively.

The most occupied BS-1 contained 43 docking poses (Figure 2b). This region included all ten docked ligands and also exhibited the lowest calculated binding energy score for acarbose (−7.3 kcal/mol). Based on comparative structural analyses, the BS-1 corresponds to the space of the putative active-site cleft, specifically its probable plus subsites (Figure 1), as predicted based on comparison with families GH8 [3,25,26,27,28,29,30] and GH48 [4,31,32,33,34,35,36,37,38,39]. The dominance of this region may partly explain the original observations by Ficko-Blean et al. [2], where CPF_2247 showed low efficiency in the hydrolysis of longer maltooligosaccharides and even the lack of activity towards substrates shorter than maltopentaose.

The second most occupied region was the BS-2 with 17 docking poses (i.e., ~2.5-fold less than the BS-1). This region showed rather a clear preference for chosen cyclic dextrins, in the order from β-cyclodextrin (5 poses) via γ-cyclodextrin (4 poses) to α-cyclodextrin (3 poses). However, maltohexaose (2 poses), maltotetraose (2 poses), and maltose (1 pose) were also recorded there (Figure 2c). The BS-2, like the BS-1, is also located in the region of the putative active-site cleft, but in the zone corresponding to the minus subsites (Figure 1), as again predicted based on comparison with families GH8 [3,25,26,27,28,29,30] and GH48 [4,31,32,33,34,35,36,37,38,39].

It should be pointed out that the remaining five BSs were positioned outside the predicted active-site cleft (Figure 2a). The BS-3 had 13 poses and spanned the region of the loop connecting the helices α8 and α9, partially involving also the C-terminus of the helix α8 and the N-terminus of the helix α9, including even the C-terminal part of the helix α7. This region was preferred by linear maltooligosaccharides (maltose to maltopentaose), namely maltose, maltotriose, and maltoheptaose (each per 1 pose), maltotetraose (2 poses), and maltopentaose (3 poses). In the BS-4, eight docking poses were seen; the site being located at the entrance to the cavity of the assumed catalytic (α/α)_6_-barrel (formed by the internal helices α2, α4, α6, α8, α10, and α12 [2]). Two poses for maltotriose and α-cyclodextrin, as well as one pose with maltopentaose, β-cyclodextrin, and acarbose, were observed in this region. The BS-5, located near the BS-4, but closer to the loop regions of helices α1, α2, and α3, and also α12, contained 6 poses. This region seemed to be particularly preferred by longer maltooligosaccharides, especially by maltoheptaose having 3 poses (vs maltohexaose with only 1 pose); although one pose was noted also with maltose and maltotriose (Figure 1c). Finally, both the BS-6 with two poses and BS-7 with only a single pose represented less represented binding regions (Figure 1b). While the BS-6 showed specificity for maltose, the single pose identified in the BS-7 was occupied by acarbose (Figure 1c).

From an energy point of view, the most stable interaction was observed for acarbose in BS-1 (−7.3 kcal/mol), where a hydrogen bond with residue Asp358 was identified (Figure 2c). On the other side, the least favorable binding energy score was recorded for maltose, varied between −5.7 and −5.1 kcal/mol (throughout all seven BSs), which is probably due to its smaller molecular size and thus limited range of possible contacts in the active-site region.

### 3.4. Targeted-Docking Trials

Based on the results of blind docking, which showed that 66.6% of the docking poses were localized in the predicted active-site cleft, targeted-docking trials were subsequently undertaken. Focusing on the joined area of BS-1 and BS-2 from blind docking, one hundred new docking poses were created and analyzed in terms of binding energy scores and interaction profiles.

**Figure 3:**
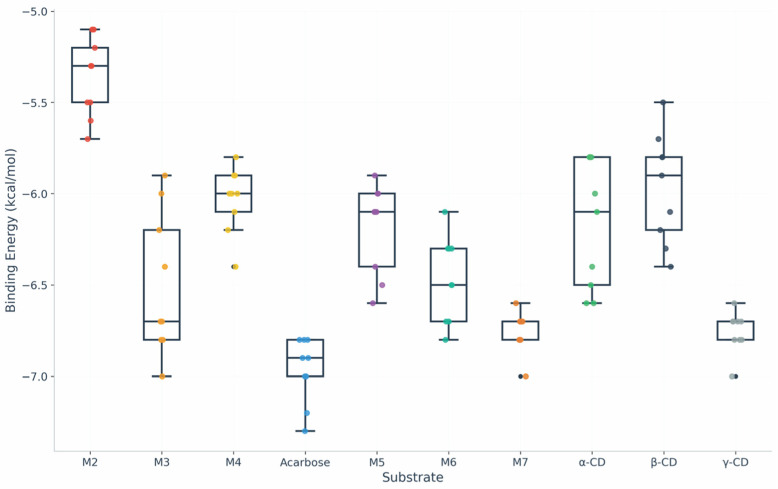
Distribution of binding energy scores (kcal/mol) for maltooligosaccharides (from maltose to maltopentaose), cyclodextrins (α-, β-, and γ-cyclodextrins), and acarbose obtained by targeted docking. Acarbose exhibited the lowest predicted binding energy scores among the analyzed ligands.

The binding energy scores differed significantly for different groups of ligands (Figure 3). The best values, i.e., the lowest binding energy scores, were observed for acarbose (on average −7.03 kcal/mol), which also demonstrated the most stable binding profiles across all poses. Its values, moreover, varied only in a relatively narrow range from −7.5 to −6.8 kcal/mol (Figure 3).

In the case of linear maltooligosaccharides, slightly higher values of binding energy scores were observed. On average, they ranged from −6.76 kcal/mol for maltoheptaose (the best) to −5.55 kcal/mol for maltose (the least), with maltotriose (−5.79 kcal/mol), maltotetraose (−5.89 kcal/mol), maltopentaose (−5.89 kcal/mol), and maltohexaose (−5.85 kcal/mol) in between, all four showing very similar values without significant differences.

In a previous study [17], maltose was docked to both GH126 representative members, the assumed amylolytic enzyme CPF_2247 [2] and EPS-specific hydrolase PssZ [6]. The docking was focused on the presumable catalytic proton donor, i.e., Glu84 (in CPF_2247) and Glu51 (in PssZ) from the putative −1 and +1 subsites. In line with the results of the present study, both maltose complexes were characterized by rather less favorable binding energy scores of −0.54 kcal/mol (for CPF_2247) and −1.07 kcal/mol (for PssZ). However, and interestingly, besides the predicted catalytic glutamic acid (Glu84 in CPF_2247), as the other functionally important (active-site) residues, Asp136, Tyr194, and Arg139 (CPF_2247 numbering) were found to be involved in hydrogen-bond contacts with maltose [17].

Obviously, maltoheptaose with a comparatively lower binding energy score (Figure 3) may thus represent the most potential maltooligosaccharide substrate. This seems to be in agreement with the preliminary experimental biochemical characterization of the assumed amylolytic enzyme CPF_2247 from *C. perfringens* [2].

The three remaining tested ligands, the cyclic dextrins, revealed average binding energy scores less favorable than acarbose and maltoheptaose—α-cyclodextrin: −6.16 kcal/mol, β-cyclodextrin: −5.96 kcal/mol, and γ-cyclodextrin: −5.97 kcal/mol. Similar to acarbose, all three ligands also showed a relatively narrow range of values across the individual docked poses (Figure 3).

Overall, the results have indicated that acarbose and maltoheptaose exhibit the strongest binding energy scores to CPF_2247, while all three cyclodextrins and most linear maltooligosaccharides fall in a less favorable energy range.

Clustering analysis showed that the majority of ligands adopted recurrent binding modes within the predicted substrate-binding cleft. Acarbose, maltose, maltotetraose, and β-cyclodextrin produced highly populated dominant clusters comprising most docking poses, whereas maltotriose, maltohexaose, γ-cyclodextrin, and, to a lesser extent, maltoheptaose and maltopentaose generated multiple, partially overlapping clusters distributed along the cleft. This behavior suggests a greater positional flexibility of these ligands within the elongated cleft. Isolated outlier poses were occasionally observed outside the dominant clusters. Nevertheless, the highest-ranked docking pose always belonged to the dominant cluster or to one of the major clusters, indicating that the selected conformations are representative of the most frequently sampled binding modes.

The individual complexes exhibited a variable number of hydrogen bonds formed between the protein and a ligand, ranging from zero to seven interactions; the complexes with three hydrogen bonds being observed most frequently. No correlation was, moreover, seen between the number of hydrogen bonds and the calculated binding energy score.

**Figure 4:**
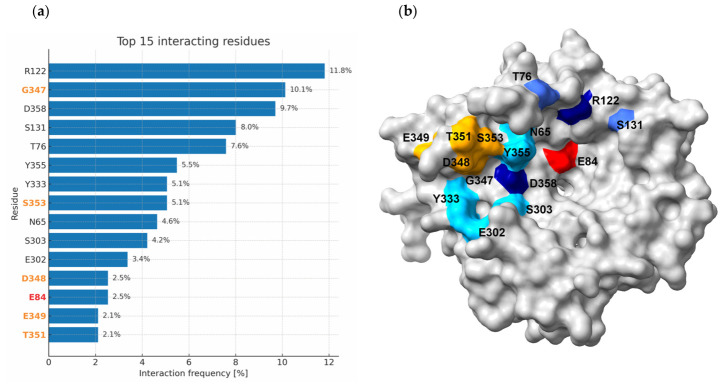
Representation of the 15 most frequently occurring residues (from a total of 37 interacting residues) involved in hydrogen bond formation during targeted docking. (**a**) Graph showing the interaction frequency of individual residues. Residues highlighted in orange belong to the α11–α12 loop (discussed in text), while the Glu84 (highlighted in red) emphasizes the predicted catalytic residue. (**b**) Surface representation of the 15 residues in CPF_2247 ([2]; PDB code: 3REN).

In total, 37 amino acid residues were identified to be involved in hydrogen-bond formation (Table S2). As shown in Figure 4, the most frequently occurring residue was Arg122 (present in the CSR-2 [17,18,19]). Its contribution covered 11.8% of all hydrogen bonds observed in all 100 targeted docking poses. The high frequency of Arg122 could be to some extent interpreted by its position in the CSR-2, where it was found conserved in ~50% of 1665 studied sequences and in an additional ~25% substituted by a lysine [18]. For example, the corresponding position in the family GH8 is more promiscuous (less arginine or lysine), whereas in GH48, that position is occupied almost exclusively by leucine or isoleucine [18].

Among the potential catalytic and/or functionally important residues, interactions were also observed with Glu84, Asp136, and Tyr194 (Table S2) located in CSR-1, CSR-3, and CSR-5, respectively [17]. Whereas the Glu84 occurred predominantly in complexes with β-cyclodextrin (3 out of 6 identified interactions), Asp136 was found when CPF_2247 docked with α-cyclodextrin (2 out of 3 poses). The third potentially functional residue, the Tyr194 [2], was involved only in a single complex with β-cyclodextrin (Table S2).

**Figure 5:**
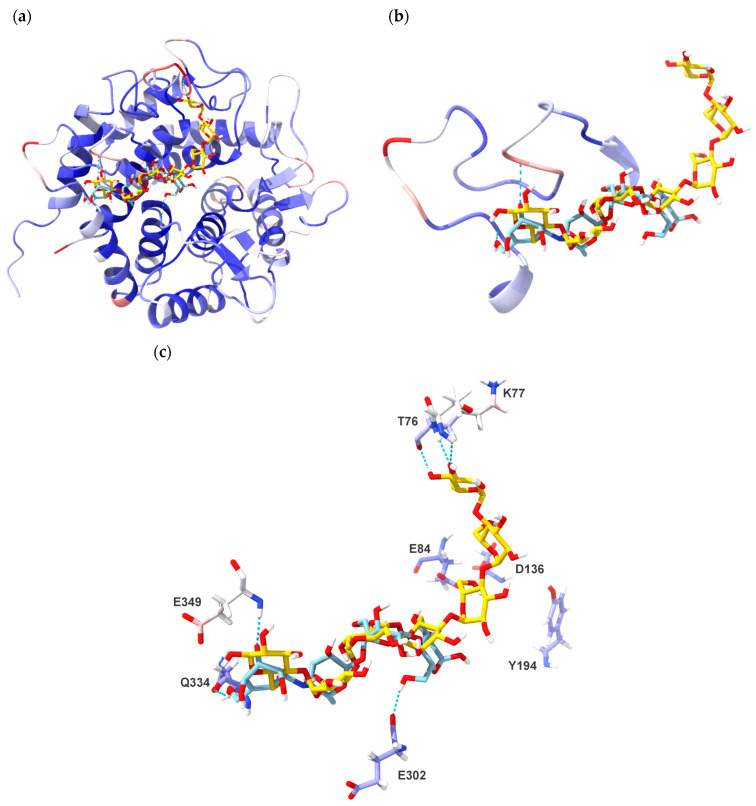
Structure of the assumed amylolytic enzyme CPF_2247 from *C. perfringens* ([2]; PDB code: 3REN) in complex with docked acarbose (blue; −7.5 kcal/mol) and maltoheptaose (yellow; −7.2 kcal/mol), representing the lowest predicted binding energy scores obtained by targeted docking. The protein structure is coloured according to the B-factor to highlight regions of structural flexibility. (**a**) Overall structure showing the docked ligands and hydrogen bond interactions. (**b**) Close-up view of the α11–α12 loop (consisting of twenty residues: 335–354) present in 22.4% of docking poses involving linear α-glucans during targeted docking. (**c**) Close-up view of both ligands with side-chains of residues involved in making the hydrogen bond contacts: (i) acarbose—Glu302, Gln334, and Glu349; and (ii) maltoheptaose—Thr76 and Lys77. The three residues best corresponding with the most important active-site residues of both GH8 and GH48 representatives—Glu84, Asp136, and Tyr194—are also displayed. If the Glu84 could be considered the assumed general acid (i.e., necessarily located closer to ligands), then the acarbose would occupy the subsites from +2 to +5, whereas the maltoheptaose would occupy the subsites from −2 to +5 (cf. with Figure 1a,b).

The spatial distribution of interacting residues confirmed the preferential localization of the binding in the region of the predicted plus subsites. Of the fifteen most frequently occurring residues involved in the hydrogen-bond forming (Figure 4), five—i.e., Gly347, Ser353, Asp348, Glu349, and Thr351—were identified to be responsible for stabilizing the two most favorable complexes. The two ligands bound in the CPF_2247 structure, i.e., acarbose and maltoheptaose with binding energy scores of −7.5 kcal/mol and −7.2 kcal/mol, respectively, are shown in Figure 5. However, the three presumable active-site residues, i.e., Glu84, Asp136, and Tyr194, are positioned too distantly for making interactions required for catalytic or binding residues (e.g., the carboxylic group of the Glu84 lies ~7.5–10.0 Å from the potential contact atoms of maltopentaose).

Analysis of B-factors in the region of residues 335–354 (the α11–α12 loop) revealed an alternation of more flexible and more rigid loop segments. In particular, residues Asn337-Asp339 and Asp348-Lys350 showed increased mobility with the side-chain B-factors values of up to 42.84 Å^2^. On the other hand, residues Leu342-Phe346 exhibited a low B-factor (approximately 12–18 Å^2^), indicating their higher structural stability.

**Figure 6:**
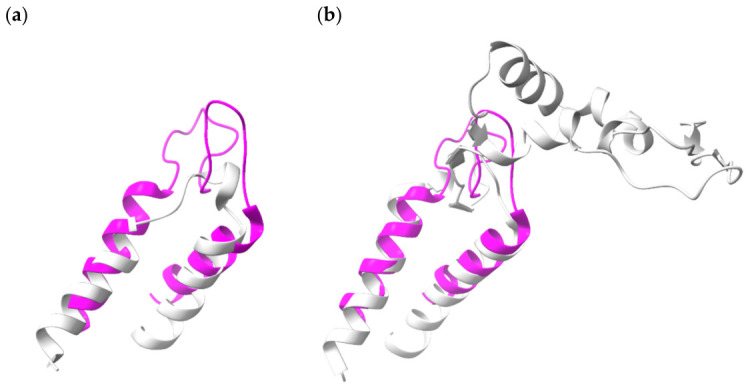
Comparison of the α11–α12 loop in families GH126, GH8, and GH48. (**a**) Superposition of the GH126 α11–α12 loop of the putative amylolytic enzyme CPF_2247 from *C. perfringens* ([2]; PDB code: 3REN; magenta) with the corresponding loop of the GH8 endo-β-1,4-glucanase CelA from *A. thermocellus* ([3]; PDB code: 1KWF; grey). (**b**) Superposition of the GH126 α11–α12 loop from CPF_2247 (magenta) with the corresponding loop of the GH48 enzyme cellobiohydrolase CelS from *A. thermocellus* ([4]; PDB code: 1L2A; grey).

It should be pointed out that the five residues mentioned above are located in a flexible loop between helices α11 and α12 of the (α/α)_6_-barrel. That loop could contribute to an important role the five residues may play in GH126 ligand recognition. Remarkably, the α11–α12 loop could represent a unique structural feature distinguishing the family GH126 from members of both families GH8 and GH48. The corresponding loop in GH8 and GH48 is substantially shorter and longer, respectively (Figure 6). Interestingly, the α11–α12 loop was as long as 16–20 residues in 148 of 198 analyzed GH126 sequences (i.e., almost in 75%) [18]. Nevertheless, since this is only a structural observation, considering the involvement of the α11–α12 loop in the eventual function and activities of the family GH126 members should currently be treated with caution since it requires experimental verification.

Finally, it should be taken into account that there is no unambiguous knowledge in the family GH126 concerning either the catalytic machinery, active-site architecture, exact enzyme specificity, or even the employed reaction mechanism. Therefore, the docking results of this study cannot represent a more conclusive proof of any of these, in fact, unknown GH126 attributes. That is in contrast with evidently more feasible docking studies performed in a related family, GH8 of the clan GH-M [27,63], where there is a wealth of structure/function information.

## 4. Conclusions

The performed structural and docking analyses provided additional insight into the relationship between the family GH126 and the GH-M clan families GH8 and GH48. Although GH126 members share the conserved (α/α)_6_-barrel fold and possess correspondences with several functionally important residues of GH8 and GH48 enzymes, their putative active-site architecture differs considerably and forms a wider open cleft. Structural comparisons indicated the eventual presence of potential catalytic machinery; some residues appear shifted in comparison with their counterparts from GH8 and GH48. Molecular docking analyses showed that most ligands preferentially occupied the predicted active-site region. Among the tested ligands, acarbose and maltoheptaose exhibited the most favorable binding energy scores, suggesting a possible preference of CPF_2247 for longer maltooligosaccharides. Several residues located in the flexible α11-α12 loop of the (α/α)_6_-barrel were identified as potentially important for ligand stabilization and substrate recognition. The combination of structural similarity to inverting β-glucanases together with the unusual substrate specificity of GH126 members further supports the idea that this family represents a rather atypical group of glycoside hydrolases. Overall, the obtained results provide a basis for future experimental studies focused on recognizing the catalytic mechanism and substrate specificity of GH126 enzymes.

## Figures and Tables

**Table 1:** List of tertiary structures involved in comparative analysis ^a^.

Family	Enzyme	PDB	Occupied Subsites	Mutants	Ref.
GH8	Endo-β-1,4-glucanase CelA	1KWF	−3, −2, −1, +1, +2	E95Q	[3]
	Endo-β-1,4-glucanase EbBssZ	7F82	−4, −3, −2, −1, +1, +2	D242A	[25]
	Endo-β-1,4-glucanase BcsZ	3QXQ	−5, −4, −3, −2, −1	E55Q	[26]
	Endo-β-1,4-xylanase Xyl	2B4F	−3, −2, −1, +1, +2	D144A	[27]
	Reducing-end xylan exo-β-1,4-xylosidase Rex	1WU6	−2, −1	E70A	[28]
	Reducing-end xylan exo-β-1,4-xylosidase RexA	6TOW	−3, −2, −1, +1	E70A	[29]
	Xylanase TtGH8	6G0N	−3, −2, −1, +1, +2	D281N	[30]
GH48	Exoglucanase ExgS	4XWN	−6, −5, −4, −3, −2, +1, +2	WT	[31]
	Cellobiohydrolase CelS	1L2A	−7, −6, −5, −4, −3, −2, +1, +2	WT	[4]
	Processive endocellulase Cel48F	1F9D	−7, −6, −5, −4, −3, −2, −1, +1, +2	E55Q	[32]
	Processive cellulase CelB	7KW6	−5, −4, −3, −2, +1, +2	WT	[33]
	Exoglucanase CelB	5CVY	−7, −6, −5, −4, −3, −2, +1, +2	WT	[34]
	UP12_08095	5VMA	−7, −6, −5, −4, −3, −2, +1, +2	WT	[35]
	Processive cellobiohydrolase Cbh48A	4TXT	+1, +2, +3	WT	[36]
	Multifunctional GH Cdan_2053	6D5D	+1, +2	WT	[37]
	Cellulase HCH_02465	4FUS	+1, +2	WT	[38]
	Exocellulase Cel48A	4JJJ	−7, −6, −5, −4, −3, −2, +1, +2	D224N	[39]

^a^ Occupied carbohydrate-binding sites in terms of the accepted nomenclature [40]. WT, wild-type.

**Table 2:** The list of ligands used in docking with the CPF_2247 ^a^.

Name	PubChem ID
Maltose	CID_439341
Maltotriose	CID_439586
Maltotetraose	CID_446495
Maltopentaose	CID_13489094
Maltohexaose	CID_5288409
Maltoheptaose	CID_13908996
α-Cyclodextrin	CID_444913
β-Cyclodextrin	CID_444041
γ-Cyclodextrin	CID_5287407
Acarbose	CID_41774

^a^ The CPF_2247, i.e., the assumed GH126 amylolytic enzyme from *C. perfringens* ([2]; PDB code: 3REN); in docking trials, the chain A was used.

**Table 3:** Comparison of GH8 and GH48 enzyme-ligand complex structures with available structures from the GH126 family (3REN and 6R2M) ^a^.

Family	Enzyme	PDB	3REN		6R2M	
			Cα	RMSD	Cα	RMSD
GH8	Endo-β-1,4-glucanase CelA	1KWF	196	1.93	233	1.92
	Endo-β-1,4-glucanase EbBssZ	7F82	238	2.00	227	2.09
	Endo-β-1,4-glucanase BcsZ	3QXQ	202	1.74	225	2.07
	Endo-β-1,4-xylanase Xyl	2B4F	210	1.73	218	2.15
	Reducing-end xylan exo-β-1,4-xylosidase Rex	1WU6	229	2.13	213	1.94
	Reducing-end xylan exo-β-1,4-xylosidase RexA	6TOW	224	1.97	221	2.03
	Xylanase TtGH8	6G0N	230	2.21	219	2.02
GH48	Exoglucanase ExgS	4XWN	253	2.00	240	2.00
	Cellobiohydrolase CelS	1L2A	201	2.01	235	1.96
	Processive endocellulase Cel48F	1F9D	264	1.88	243	1.97
	Processive cellulase CelB	7KW6	269	2.15	269	1.96
	Exoglucanase CelB	5CVY	275	1.93	237	1.91
	UP12_08095	5VMA	262	2.17	240	1.93
	Processive cellobiohydrolase Cbh48A	4TXT	258	1.92	239	1.95
	Multifunctional GH Cdan_2053	6D5D	253	2.00	238	1.88
	Cellulase HCH_02465	4FUS	260	1.93	244	1.99
	Exocellulase Cel48A	4JJJ	259	1.87	237	1.92

^a^ Each overlap is characterized by the number of corresponding Cα-atoms (left column) identified in a particular structural overlap and the relevant RMSD value in Å (right column).

## Data Availability

The original data presented in this study are included in the article/Supplementary Material. Further inquiries can be directed to the corresponding author.

## Supplementary Materials

The following supporting information can be downloaded at: https://www.mdpi.com/article/10.3390/molecules31132273/s1. Table S1: Best structural matches identified by FoldSeek for the amylolytic enzyme CPF_2247 and the EPS-specific hydrolase PssZ. Table S2: Complete list of residues involved in hydrogen bond interactions observed during targeted docking.

## Abbreviations

The following abbreviations are used in this manuscript:

BS	Binding site
CAZy	Carbohydrate-Active enzyme database
CSR	Conserved sequence region
EPS	Exopolysaccharide
GH	Glycoside hydrolase
PDB	Protein Data Bank
RMSD	Root mean square deviation
