# Supplementary material for: In Silico Study of Potential Binding Sites of the Family GH126 Enzyme CPF_2247 from Clostridium perfringens Using Structural Comparison and Molecular Docking Methods
Source: Molecules. 2026 Jun 29;31(13):2273. doi: 10.3390/molecules31132273 (PMC13363691; doi:10.3390/molecules31132273)
Supplement: Supplementary file 1 [file molecules-31-02273-s001.zip › Janecek_S_Table_S1-SI_Amylases_2nd_Ed.pdf]

Table S1. Best structural matches identified by FoldSeek for the amylolytic enzyme CPF_2247 and the EPS-specific hydrolase PssZ.[a]

3REN

Family	Enzyme	Organism of origin	PDB	Ligand[c]	Mutation	SI (%)	E-value	Score	Query position	Target position
GH126	CPF_2247	*Clostridium perfringens*	3ren_A			100	1.40E-60	2812	1-337 (337)	1-337 (337)
GH126	CPF_2247	*Clostridium perfringens*	3ren_B			99.1	3.44E-54	2496	2-336 (337)	1-332 (332)
GH126	PssZ	*Listeria monocytogenes*	6r2m_B			19.5	1.30E-14	538	13-335 (337)	13-305 (310)
GH126	PssZ	*Listeria monocytogenes*	6r2m_A			18.8	1.30E-14	537	13-333 (337)	13-303 (311)
GH8	CelA	*Acetivibrio thermocellus*	1is9_A			19.9	1.51E-12	417	8-331 (337)	21-344 (358)
GH8	CelA	*Acetivibrio thermocellus*	1cem_A			19.4	4.64E-12	399	8-332 (337)	26-349 (363)
GH8	CelA	*Acetivibrio thermocellus*	1kwf_A	C5	E95Q	19.8	1.05E-11	391	8-332 (337)	25-349 (363)
GH8	TE10_01090	*Raoultella ornithinolytica*	5czl_A			15.4	9.46E-11	359	13-335 (337)	10-313 (315)
GH8	Cel10	*Klebsiella pneumoniae*	5gy3_A			14.1	6.96E-11	358	13-336 (337)	4-310 (310)
GH8	BcsZ	*Escherichia coli*	3qxf_C			10.7	1.35E-10	354	13-331 (337)	4-317 (337)
GH8	VFA0882	*Aliivibrio fischeri*	5cd2_A			15.6	8.54E-11	354	13-335 (337)	4-327 (345)
GH8	Lic8H	*Paenibacillus* sp. X4	5xd0_B			16.4	2.91E-10	345	8-334 (337)	29-373 (378)
GH8	CMCax	*Komagataeibacter xylinus*	1wzz_A			15.7	1.28E-09	324	9-335 (337)	5-314 (319)
GH8	BcsZ	*Pseudomonas putida*	4q2b_C			11.6	8.08E-10	321	13-331 (337)	5-322 (343)
GH8	BcsZ	*Enterobacter* sp. CJF-002	7f81_C			13.2	1.28E-09	316	13-335 (337)	5-321 (338)
GH8	BcsZ	*Enterobacter* sp. CJF-002	7f82_D	C4 + C2	D242A	13.8	5.65E-10	316	13-331 (337)	5-318 (338)
GH8	Rex	*Halalkalibacterium halodurans*	1wu5_A	X1		17.4	6.59E-10	316	8-336 (337)	22-368 (376)
GH8	Rex	*Halalkalibacterium halodurans*	2dro_A		D263C	16.6	3.21E-09	314	46-336 (337)	61-368 (376)
GH8	Chok	*Bacillus cereus*	1v5d_A			15.6	2.90E-09	312	8-335 (337)	29-381 (386)
GH8	Rex	*Halalkalibacterium halodurans*	2drr_A		D263N	16.5	2.75E-09	311	46-336 (337)	61-368 (376)
GH8	BcsC	*Escherichia coli*	3qxq_D	C5	E55Q	11.8	2.75E-09	309	13-335 (337)	5-321 (338)
GH8	Xyl	*Pseudoalteromonas haloplanktis*	2b4f_A	X5	D144A	15.6	1.93E-09	309	8-336 (337)	32-394 (404)
GH8	CMCax	*Komagataeibacter sucrofermentans*	6vc5_A			17.5	1.57E-09	308	9-335 (337)	2-307 (318)
GH8	Rex	*Halalkalibacterium halodurans*	1wu6_A	X2		16.9	5.08E-09	307	46-336 (337)	61-368 (376)
GH8	Rex	*Halalkalibacterium halodurans*	1wu4_A			15.9	5.08E-09	303	8-336 (337)	25-366 (374)
GH8	Xyl	*Pseudoalteromonas haloplanktis*	1h14_A		D144N	15.4	5.35E-09	300	46-336 (337)	74-398 (404)
GH8	Rex	*Halalkalibacterium halodurans*	2drq_A		D263G	16.6	5.63E-09	298	46-336 (337)	62-369 (377)
GH8	Rex	*Halalkalibacterium halodurans*	3a3v_A		Y198F	16.5	9.38E-09	297	46-336 (337)	61-368 (376)
GH8	TtGH8	*Teredinibacter turnerae*	6g09_A			13.7	7.65E-09	297	8-336 (337)	20-388 (392)
GH8	TtGH8	*Teredinibacter turnerae*	6g0n_A	X5	D281N	13.2	8.05E-09	296	8-336 (337)	24-388 (392)
GH8	Rex	*Halalkalibacterium halodurans*	2drs_A		D263S	16.3	8.47E-09	294	46-336 (337)	64-371 (379)
GH8	PbRex8	*Paenibacillus barengoltzii*	5yxt_C			14.9	1.92E-08	287	8-336 (337)	30-370 (379)
PF07221	XfasM23_1239	*Xylella fastidiosa*	3gt5_A			8.4	7.27E-09	286	8-334 (337)	12-384 (391)
PF07221	Cellobiose 2-epimerase	*Caldibacillus thermoamylovorans*	5zhb_B			12.2	1.41E-08	283	7-335 (337)	8-377 (379)
GH8	Xyl	*Pseudoalteromonas haloplanktis*	1xwq_A	X3	E78Q	16.7	1.93E-09	283	8-337 (337)	31-402 (404)
GH8	Rex8A	*Paenibacillus barcinonensis*	6srd_B	X1		14.1	4.36E-09	281	10-337 (337)	20-374 (377)
GH8	Xyl	*Pseudoalteromonas haloplanktis*	1h13_A			16	4.59E-09	278	8-337 (337)	32-402 (404)
GH8	Xyl	*Pseudoalteromonas haloplanktis*	1xwt_A		D281N	16	5.93E-09	277	8-337 (337)	32-402 (404)
PF07221	Cellobiose 2-epimerase	*Caldicellulosiruptor saccharolyticus*	4z4l_A			10.8	5.90E-08	271	9-337 (337)	16-392 (392)
GH8	Rex8A	*Paenibacillus barcinonensis*	6sud_B	X1	L320A	15.3	3.04E-08	270	2-336 (337)	17-369 (377)
PF07221	Marme_2490	*Marinomonas mediterranea*	5x32_B			9.5	4.35E-08	268	8-334 (337)	13-386 (391)
PF07221	Cellobiose 2-epimerase	*Caldicellulosiruptor saccharolyticus*	8wbv_A	Man	H247F	11.3	5.33E-08	267	10-335 (337)	17-390 (391)
PF07221	Mannose-2-epimerase	*Runella slithyformis*	8h1l_D	SOR		9.1	3.37E-08	267	7-335 (337)	9-415 (419)
PF07221	Glucose-2-epimerase	*Runella slithyformis*	8h1l_A	SOR		9	4.13E-08	266	9-335 (337)	17-414 (418)
GH8	Rex8A	*Paenibacillus barcinonensis*	6trh_C			13.9	1.73E-08	265	2-336 (337)	22-378 (385)
PF07221	Glucose-2-epimerase	*Runella slithyformis*	8h1m_A		D254A	9.1	6.21E-08	263	7-335 (337)	9-418 (421)
PF07221	Marme_2490	*Marinomonas mediterranea*	5x32_A			9.7	9.35E-08	261	8-332 (337)	14-373 (380)
PF07221	Cellobiose 2-epimerase	*Caldicellulosiruptor saccharolyticus*	7d5g_A	Glc-Fru		10.1	1.15E-07	260	8-335 (337)	6-388 (389)
PF07221	YihS	*Salmonella enterica* subsp. enterica[b]	2zbl_C	Man	H248A	10.9	3.54E-08	257	8-332 (337)	15-391 (417)
GH8	Rex8A	*Paenibacillus barcinonensis*	6shy_B		L320A, H321S	15.4	4.35E-08	256	2-337 (337)	17-374 (377)

6R2M

Family	Enzyme	Organism of origin	PDB	Ligand[c]	Mutation	SI (%)	E-value	Score	Query position	Target positions
GH126	PssZ	*Listeria monocytogenes*	6r2m_A			100	4.83E-61	2640	1-311 (311)	1-311 (311)
GH126	PssZ	*Listeria monocytogenes*	6r2m_B			99.6	6.71E-58	2474	1-311 (311)	1-310 (310)
GH126	CPF_2247	*Clostridium perfringens*	3ren_B			19.5	3.10E-16	565	13-305 (311)	12-332 (332)
GH126	CPF_2247	*Clostridium perfringens*	3ren_A			19	5.99E-16	550	13-304 (311)	13-334 (337)
GH8	BcsZ	*Pseudomonas putida*	4q2b_C			11.8	4.81E-09	287	17-302 (311)	9-323 (343)
GH8	CMCax	*Komagataeibacter xylinus*	1wzz_A			14.4	1.79E-09	281	7-303 (311)	1-312 (319)
GH8	BcsC	*Escherichia coli*	3qxq_D	C5	E55Q	9.7	1.44E-08	275	17-302 (311)	9-319 (338)
GH8	BcsZ	*Enterobacter* sp. CJF-002	7f82_D	C4 + C2	D242A	11	1.79E-08	273	15-302 (311)	7-319 (338)
GH8	CelA	*Acetivibrio thermocellus*	1is9_A			12.1	2.49E-08	272	14-302 (311)	26-345 (358)
GH8	Cel10	*Klebsiella pneumoniae*	5gy3_A			15.1	1.36E-08	270	15-304 (311)	5-306 (310)
GH8	VFA0882	*Aliivibrio fischeri*	5cd2_A			16.6	2.78E-08	266	17-302 (311)	8-324 (345)
GH8	CelA	*Acetivibrio thermocellus*	1cem_A			12.7	7.45E-08	256	15-303 (311)	32-351 (363)
GH8	BcsC	*Escherichia coli*	3qxf_C			12.3	1.70E-08	252	17-302 (311)	8-318 (337)
GH8	BcsZ	*Enterobacter* sp. CJF-002	7f81_C			12.8	5.36E-08	252	15-303 (311)	7-317 (338)
PF07221	Mannose-2-epimerase	*Runella slithyformis*	8h1l_D	SOR		10	7.87E-09	246	14-304 (311)	21-414 (419)
GH8	Lic8H	*Paenibacillus* sp. X4	5xd0_C			13.2	3.86E-08	239	1-305 (311)	14-375 (378)
PF07221	Glucose-2-epimerase	*Runella slithyformis*	8h1m_A		D254A	9.4	7.87E-09	236	14-305 (311)	21-421 (421)
PF07221	Mannose-2-epimerase	*Runella slithyformis*	8h1l_A			10.3	8.79E-09	235	15-304 (311)	23-413 (418)
GH8	Rex	*Halalkalibacterium halodurans*	1wu4_A			14.4	8.32E-08	234	13-303 (311)	23-364 (374)
GH8	CMCax	*Komagataeibacter sucrofermentans*	6vc5_A			15.6	3.10E-07	233	15-306 (311)	7-312 (318)
GH8	CelA	*Acetivibrio thermocellus*	1kwf_A	C5	E95Q	12.5	2.63E-07	232	14-303 (311)	31-351 (363)
GH8	Rex	*Halalkalibacterium halodurans*	2drs_A		D263S	13.8	1.89E-07	227	13-303 (311)	26-369 (379)
GH8	Xyl	*Pseudoalteromonas haloplanktis*	2b4f_A	X5	D144A	12.4	1.60E-07	225	10-303 (311)	26-389 (404)
GH8	Rex	*Halalkalibacterium halodurans*	2dro_A		D263C	12.8	9.28E-08	224	13-303 (311)	23-366 (376)
GH8	TE10_01090	*Raoultella ornithinolytica*	5czl_A			14.9	3.65E-07	221	14-304 (311)	11-312 (315)
GH8	Rex	*Halalkalibacterium halodurans*	1wu6_A	X2		13.1	6.68E-08	221	13-303 (311)	23-366 (376)
GH8	Rex	*Halalkalibacterium halodurans*	3a3v_A			13.3	2.00E-07	220	13-303 (311)	23-366 (376)
GH8	Rex	*Halalkalibacterium halodurans*	2drr_A		D263N	12.8	2.63E-07	220	38-303 (311)	61-366 (376)
PF07221	Glucose-2-epimerase	*Runella slithyformis*	8h1k_B			9.6	1.89E-08	220	14-305 (311)	21-421 (421)
GH8	Rex	*Halalkalibacterium halodurans*	2drq_A		D263G	14.1	2.63E-07	215	13-303 (311)	26-363 (377)
GH8	Xyl	*Pseudoalteromonas haloplanktis*	1xwq_A	X3	E78Q	10.3	4.08E-07	215	10-303 (311)	26-389 (404)
GH8	Rex8A	*Paenibacillus barcinonensis*	6shy_B		L32A, H321S	11.8	3.65E-07	214	13-303 (311)	28-367 (377)
PF07221	Cellobiose 2-epimerase	*Caldicellulosiruptor saccharolyticus*	7d5g_A	Glc-Fru		10.4	9.80E-08	213	13-303 (311)	10-388 (389)
PF07221	Cellobiose 2-epimerase	*Caldicellulosiruptor saccharolyticus*	4z4l_A			11.1	2.11E-07	211	15-305 (311)	22-392 (392)
PF07221	Cellobiose 2-epimerase	*Caldicellulosiruptor saccharolyticus*	8wbv_A	Man	H247F	10.1	2.63E-07	209	13-303 (311)	12-390 (391)
GH8	Rex8A	*Paenibacillus barcinonensis*	6srd_B	X1		12.5	1.04E-06	207	13-302 (311)	28-365 (377)
GH8	Xyl	*Pseudoalteromonas haloplanktis*	1h14_A		D144N	12.4	9.80E-07	206	10-303 (311)	26-389 (404)
GH8	Xyl	*Pseudoalteromonas haloplanktis*	1h13_A			12.1	1.15E-06	203	10-303 (311)	26-389 (404)
PF07221	Cellobiose 2-epimerase	*Rhodothermus marinus*	3wkf_A			9.4	2.78E-07	202	4-303 (311)	1-395 (407)
GH8	TtGH8	*Teredinibacter turnerae*	6g0n_A	X5	D281N	11.7	3.65E-06	200	22-302 (311)	49-380 (392)
GH8	Rex8A	*Paenibacillus barcinonensis*	6sud_B	X1	L32A	13.7	2.23E-06	199	21-302 (311)	45-365 (377)
PF07221	Marme_2490	*Marinomonas mediterranea*	5x32_B			9.7	2.00E-06	198	14-303 (311)	19-386 (391)
GH8	Xyl	*Pseudoalteromonas haloplanktis*	1xwt_A		D281N	12.1	2.00E-06	198	10-303 (311)	26-389 (404)
PF07221	Cellobiose 2-epimerase	*Ruminococcus albus*	3vw5_C			9.2	4.31E-07	195	13-305 (311)	14-387 (389)
PF07221	Cellobiose 2-epimerase	*Caldibacillus thermoamylovorans*	5zhb_B			9.3	1.29E-06	194	14-303 (311)	17-373 (379)
PF07221	Marme_2490	*Marinomonas mediterranea*	5x32_A			10	2.63E-06	194	14-303 (311)	20-375 (380)
GH8	C812_03928	*Paenibacillus barengoltzii*	5yxt_C			13.2	1.09E-06	193	12-302 (311)	26-366 (379)
GH8	TtGH8	*Teredinibacter turnerae*	6g09_A			11.4	7.87E-06	190	22-302 (311)	49-380 (392)
PF07221	Cellobiose 2-epimerase	*Caldibacillus thermoamylovorans*	5zhb_A			7.9	6.68E-06	181	14-303 (311)	17-374 (380)
PF07221	YihS	*Salmonella enterica* subsp. enterica[b]	2afa_F			9.8	2.11E-06	178	8-302 (311)	4-384 (408)

[a] Probability was in each case equal to "1". [b] *Salmonella enterica* subsp. enterica serovar Typhimurium. [c] C2, cellobiose; C4, cellotetraose; C5, cellopentaose; X1, xylose; X2, xylobiose; X3, xylotriose; X5, xylopentaose; Man, manose; SOR, sorbitol; EPE, 4-(2-hydroxyethyl)-1-piperazine ethanesulfonic acid; Glc-Fru, glucopyranosyl-β-1,4-fructose.
