# Supplementary material for: In Silico Study of Potential Binding Sites of the Family GH126 Enzyme CPF_2247 from Clostridium perfringens Using Structural Comparison and Molecular Docking Methods
Source: Molecules. 2026 Jun 29;31(13):2273. doi: 10.3390/molecules31132273 (PMC13363691; doi:10.3390/molecules31132273)
Supplement: Supplementary file 1 [file molecules-31-02273-s001.zip › Janecek_S_Table_S2_SI_Amylases_2nd_Ed.pdf]

Table S2. Complete list of residues involved in hydrogen bond interactions observed during targeted docking.[a]

Residue	Count	Occurrences	Note
Arg122	28	Acarbose_7, αCD_3, αCD_5, βCD_1, βCD_2, βCD_6, βCD_7, βCD_8, βCD_9, γCD_10, γCD_3, γCD_4, γCD_6, M3_9, M4_2, M4_6, M5_4, M5_8, M6_10, M6_3, M6_5, M6_8, M6_9, M7_1, M7_10, M7_2, M7_3, M7_6	CSR-2
Gly347	25	Acarbose_10, Acarbose_9, M2_1, M2_3, M2_6, M2_8, M2_9, M3_2, M3_3, M3_6, M3_7, M4_1, M4_5, M4_7, M4_9, M5_1, M5_6, M5_7, M6_1, M6_2, M6_3, M6_6, M6_9, M7_6, M7_8	loop α11-12
Asp358	23	Acarbose_1, Acarbose_3, Acarbose_5, γCD_1, M2_10, M2_4, M3_2, M3_3, M3_7, M4_10, M4_7, M4_9, M5_3, M5_4, M5_8, M6_1, M6_2, M6_6, M7_1, M7_10, M7_3, M7_7, M7_8	CSR-7
Ser131	19	αCD_1, αCD_3, αCD_5, αCD_6, αCD_9, βCD_1, βCD_3, βCD_4, βCD_5, βCD_6, βCD_8, βCD_9, γCD_10, γCD_5, γCD_6, γCD_8, M3_9, M6_10, M6_8	
Thr76	18	βCD_4, βCD_6, γCD_10, γCD_4, γCD_5, M3_9, M4_2, M4_6, M5_3, M5_8, M6_6, M6_8, M7_1, M7_10, M7_2, M7_4, M7_6, M7_7	
Tyr355	13	γCD_3, γCD_4, M2_2, M2_3, M2_5, M3_1, M3_6, M5_7, M6_9, M7_1, M7_4, M7_5, M7_6	CSR-7
Tyr333	12	Acarbose_1, Acarbose_10, M2_10, M2_3, M2_4, M2_5, M3_1, M4_9, M5_1, M5_2, M5_6, M7_6	
Ser353	12	Acarbose_2, αCD_2, αCD_7, βCD_10, M4_5, M5_1, M5_4, M5_7, M5_9, M6_2, M6_4, M6_9	loop α11-12
Asn65	11	βCD_2, βCD_6, βCD_8, γCD_1, γCD_2, γCD_6, M4_2, M4_6, M5_3, M6_3, M6_8	
Ser303	10	Acarbose_1, Acarbose_6, Acarbose_9, M3_5, M4_7, M5_10, M6_10, M6_3, M7_3, M7_4	CSR-6
Glu302	8	αCD_2, αCD_4, γCD_9, M2_9, M4_3, M4_5, M4_8, M7_5	CSR-6
Glu84	6	αCD_6, βCD_1, βCD_3, βCD_9, M3_8, M7_3	CSR-1
Asp348	6	αCD_2, αCD_γCD_9, M2_6, M5_6, M6_3, M7_2	loop α11-12
Glu349	6	Acarbose_1, Acarbose_3, M3_10, M4_10, M6_2, M6_4	loop α11-12
Thr351	5	M5_7, M6_2, M6_4, M6_9, M7_10	loop α11-12
Asp136	3	αCD_3, αCD_8, M2_7	CSR-3
Thr189	3	γCD_8, M5_5, M7_5	
Ser80	3	M6_3, M6_9, M7_3	
Asp251	2	Acarbose_8, M4_7	
Tyr180	2	αCD_9, βCD_7	
Ser129	2	γCD_8, M6_10	

Cys193	2	M3_5, M6_5	CSR-5
Gln334	2	M5_10, M5_6	
Gly347	2	M3_1, M3_2	loop α11-12
Lys77	2	M6_5, M7_2	
Gly348	1	Acarbose_4	loop α11-12
Ala288	1	Acarbose_8	
Lys350	1	αCD_2	loop α11-12
Lys235	1	αCD_10	
Tyr194	1	βCD_4	CSR-5
Asp74	1	γCD_6	
Arg139	1	M3_5	CSR-3
Phe357	1	M4_2	CSR-7
Leu253	1	M5_8	
Ser70	1	M5_9	
Trp121	1	M6_10	CSR-2
Tyr238	1	M7_5	

[a] Total number of hydrogen-bond forming residues was 37.
